# *Implementation science*: a reappraisal of our journal mission and scope

**DOI:** 10.1186/s13012-015-0240-2

**Published:** 2015-04-17

**Authors:** Robbie Foy, Anne Sales, Michel Wensing, Gregory A Aarons, Signe Flottorp, Bridie Kent, Susan Michie, Denise O’Connor, Anne Rogers, Nick Sevdalis, Sharon Straus, Paul Wilson

**Affiliations:** Leeds Institute of Health Sciences, University of Leeds, Leeds, UK; VA Center for Clinical Management Research, VA Ann Arbor Healthcare System, Ann Arbor, MI USA; School of Nursing, University of Michigan, Ann Arbor, MI USA; Radboud Institute for Health Sciences, Radboud University Medical Centre, Nijmegen, Netherlands; University of California, San Diego, La Jolla, CA USA; Norwegian Knowledge Centre for the Health Services, Oslo, Norway; School of Nursing and Midwifery, Faculty of Health and Human Sciences, Plymouth University, Plymouth, UK; Centre for Behaviour Change, Department of Clinical, Educational and Health Psychology, University College London, London, UK; School of Public Health and Preventive Medicine, Monash University, Melbourne, Australia; National Institute for Health Research Collaboration for Leadership in Applied Health Research and Care (NIHR CLAHRC) Wessex, Faculty of Health Sciences, University of Southampton, Southampton, UK; Centre for Implementation Science, Health Service and Population Research Department, King’s College, London, UK; Li Ka Shing Knowledge Institute of St Michael’s, University of Toronto, Toronto, Canada; Manchester Business School, University of Manchester, Manchester, UK

**Keywords:** Implementation research, Methodology

## Abstract

The implementation of research findings into healthcare practice has become increasingly recognised as a major priority for researchers, service providers, research funders and policymakers over the past decade. Nine years after its establishment, *Implementation Science*, an international online open access journal, currently publishes over 150 articles each year. This is fewer than 30% of those submitted for publication. The majority of manuscript rejections occur at the point of initial editorial screening, frequently because we judge them to fall outside of journal scope. There are a number of common reasons as to why manuscripts are rejected on grounds of scope. Furthermore, as the field of implementation research has evolved and our journal submissions have risen, we have, out of necessity, had to become more selective in what we publish. We have also expanded our scope, particularly around patient-mediated and population health interventions, and will monitor the impact of such changes. We hope this editorial on our evolving priorities and common reasons for rejection without peer review will help authors to better judge the relevance of their papers to *Implementation Science*.

## Editorial

The implementation of research findings into practice has become increasingly recognised as a major priority for researchers, research funders and policymakers over the past decade [1-3]. Nine years after its establishment, *Implementation Science* currently publishes over 150 articles each year. This is fewer than 30% of those submitted for publication. The majority of manuscript rejections occur at the point of initial editorial screening, frequently because we judge them to fall outside of journal scope. There are a number of common reasons as to why manuscripts are rejected on grounds of scope. However, as the field of implementation research has evolved and our journal submissions have risen, we have also, out of necessity, had to become more selective in what we publish. This partly reflects our strategy for sustainable growth: despite being an open access journal with potentially infinite space for publishing articles, we inevitably have limited editorial and reviewer resources. We therefore need to prioritise manuscripts which can potentially make significant contributions to knowledge, methodology or thinking—and aim to provide high quality support to both authors and reviewers to achieve this.

### Emerging issues and developments

Following discussions within our editorial team and consultations with our Editorial Board and Senior Advisory Board, we take this opportunity to reappraise our mission and update our scope last set out in 2012 [4,5]. Emerging issues and developments include the following:

- Extension of our field of interest, which predominantly has been evidence-based healthcare practice, to include evidence-based population health;
- Recognition of a continuum between studies of clinical or population interventions and studies of implementation interventions, and the need to clarify boundaries for journal scope;
- Prioritisation of studies which make substantial, rather than marginal, contributions to the field of implementation research.

### Our revised scope

*Implementation Science* is an open access, peer-reviewed online journal that aims to publish research relevant to promoting the uptake of research findings into healthcare practice and health policy.

Health and healthcare research constantly produce new findings—but often these are not routinely translated into practice. This loss in translation represents part of a wider problem of systemic ‘research waste’ in the production and reporting of research evidence [6,7]. Implementation research is the scientific study of methods to promote the systematic uptake of evidence-based interventions into practice and policy and hence improve health. In this context, it includes the study of influences on professional, patient and organisational behaviour in healthcare, community or population contexts.

The lack of routine uptake of research findings is strategically important for the efforts to improve health and healthcare because it places an invisible ceiling on the potential for research to enhance population outcomes. Further, it is scientifically important because it identifies the behaviour of patients, professionals, organisations and systems as key sources of variance requiring improved empirical and theoretical understanding before effective uptake can be reliably achieved.

Implementation science is an inherently interdisciplinary research area, and the journal is not constrained by any particular research method. *Implementation Science* wishes to publish articles of high scientific rigour using the most appropriate methods to produce valid, generalisable answers to research questions. As well as hosting papers describing the effectiveness and economics of implementation interventions, *Implementation Science* provides a unique home for articles describing intervention development, evaluations of processes by which effects are achieved and sustained and the role of theory relevant to implementation research. The journal is interested in publishing articles that present novel methods (particularly those that have a sound theoretical basis or offer design advantages) for studying the determinants, processes and effects of implementation interventions. We are also interested in receiving articles that report methodologically robust studies of the de-implementation of ineffective or harmful practices.

We welcome study protocols, but typically we will only consider these if the study has received ethical approval by a recognised institutional body and been through external peer review via an established national or international funding body. We do not consider protocols for systematic reviews or protocols for quantitative studies that have begun data cleaning or analysis.

We expect authors to follow relevant reporting guidelines for studies to help ensure the transparency and quality of our publications [8].

Alongside elaborating issues we are particularly interested in, we take this opportunity to address a number of common specific content and methodological matters that lead us to reject a paper submitted to *Implementation Science*.

### Is it implementation research?

We initially assess whether submitted papers deal with investigating, as opposed to conducting, implementation. We expect studies to include a clear scientific question and use an appropriate design, which can answer that question. For example, we generally reject descriptive reports of quality improvement initiatives. We aim to prioritise manuscripts that offer significant contributions to knowledge, methodology or thinking within the implementation science field.

We are interested in studies examining the implementation of evidence-based practices or policies or the de-implementation of those demonstrated to be relatively ineffective or even harmful [9]. We expect authors to include a brief summary of the existing evidence base for any practice or policy being implemented, preferably drawing upon systematic reviews and meta-analyses or rigorous primary empirical studies.

We publish studies which evaluate clearly defined implementation interventions but not studies testing novel clinical or population health interventions. We often find it difficult to judge whether evaluations of novel organisational or service delivery methods are within scope, for example, variants of collaborative care for depression often combine implementation and clinical components [10]. We therefore continue to judge such submissions on a case-by-case basis. However, we advise authors to draw upon an existing taxonomy for defining and describing interventions, such as that set out by the Cochrane Collaboration Effective Practice and Organisation of Care Group [11]. We recognise that many implementation interventions are relatively complex and may possess interacting components, target different behaviours, groups, organisational levels and outcomes, and require degrees of tailoring [12].

### Scope and boundaries related to article type

#### Theories and frameworks

We welcome studies building and advancing theories and frameworks relevant to implementation, preferably through empirical testing. There is no shortage of such frameworks and theories, many of which are poorly conceptualised or have not been tested [13]. We often receive manuscripts which set out new theories and frameworks and usually reject those which do not explicitly acknowledge or build upon existing work.

#### Systematic reviews

We publish systematic reviews, which may cover issues such as the effects of implementation interventions and influences on the uptake of evidence. We also consider other types of reviews (e.g. rapid, realist or scoping) but expect them to use systematic methods to identify studies and report methods, including strengths and limitations, as transparently as traditional systematic reviews.

#### Evaluations of implementation interventions

We expect studies that evaluate effectiveness to use rigorous experimental or quasi-experimental designs, such as randomised controlled trials or interrupted time-series analyses. We generally reject studies with poorer internal validity, such as those using uncontrolled before-after designs. We recognise that evaluations of implementation interventions can use mixed methods approaches, so that strong effectiveness evaluation designs may be accompanied by carefully designed qualitative or quantitative process evaluations which elucidate mechanisms of action, identify contextual factors that moderate their effects and provide insights into implementation processes. A considerable body of evidence about the effects of implementation interventions now exists, and we expect authors to cite existing systematic reviews or primary studies as relevant.

We receive a number of submissions reporting ‘effectiveness-implementation hybrid design’ studies. Curran et al*.* defined such studies as follows [14]:

‘An effectiveness-implementation hybrid design is one that takes a dual focus *a priori* in assessing clinical effectiveness and implementation. We propose three hybrid types: (1) testing effects of a clinical intervention on relevant outcomes while observing and gathering information on implementation; (2) dual testing of clinical and implementation interventions/strategies; and (3) testing of an implementation strategy while observing and gathering information on the clinical intervention’s impact on relevant outcomes’.

Any good clinical or health services researcher would naturally welcome research which anticipates or addresses likely future implementation issues as early as possible in the development and evaluation of clinical interventions. However, publishing earlier stage hybrid designs would detract from our mission which focuses on the implementation of interventions of demonstrated effectiveness. We are generally interested in types 2 and 3 hybrid designs with a clear justification and major element of implementation research. Therefore, we usually reject type 1 hybrid designs.

We also receive manuscripts reporting studies which investigate the effectiveness of a healthcare intervention of demonstrated effectiveness in one context and then evaluate its effects in another context (e.g. different setting or patient population). However, we usually reject such manuscripts, regarding them as essentially primary effectiveness studies with little contribution to implementation theory or research.

#### Economic evaluation

Although there is a large and growing evidence base relating to the effectiveness of implementation strategies, evidence evaluating their costs has been neglected [15]. Dissemination and implementation strategies are not without costs and compete with other healthcare activity for finite resources. We encourage submissions of empirical studies which include an economic evaluation component [16].

#### Process evaluations

We are interested in process evaluations of *implementation* interventions. We receive but are less interested in process evaluations examining the implementation (or fidelity) of clinical or population health interventions being evaluated within primary effectiveness studies. For example, a process evaluation alongside a trial randomising individual patients in order to evaluate the effectiveness of a form of counselling for depression would generally fall outside of our scope. However, a process evaluation alongside a cluster randomised trial evaluating the effects of a strategy to implement a form of counselling for depression of known effectiveness would come inside our scope.

We welcome process evaluations (qualitative, quantitative or mixed methods) which can increase understanding of a range of issues affecting the outcomes of effectiveness studies, such as intervention uptake and adherence, influence of context, mechanisms of action and unintended consequences [17]. We need to know the outcomes of the associated effectiveness study so that readers can tell whether a process evaluation aims to explain positive or null outcomes. We typically reject process evaluations that do not refer to the effectiveness study outcomes.

#### Qualitative studies

We frequently receive manuscripts using qualitative methods for data collection and analysis of implementation research. We publish those that fit our scope and meet applicable criteria for quality and validity. We usually reject qualitative studies where there are doubts whether planned data saturation has been achieved and single site case studies which limit typicality. We often receive manuscripts that report on essentially content analysis of interviews, without appropriate links to relevant theory or without contextualisation and with little reference to previous relevant qualitative studies or reviews. We routinely reject these. There is a growing body of meta-syntheses of qualitative studies, and we expect reference to those that are relevant.

#### Intervention development reports

We welcome reports of intervention development which demonstrate novel methods, provide empirical or theoretical rationale for intervention content or offer content of wider interest. We do not generally publish standalone intervention development reports with no stated intention to undertake a rigorous evaluation. We usually reject reports describing interventions that are submitted after the main evaluation results are known because of the risks of post hoc rationalisation, whereby authors may consciously or unintentionally modify intervention content in light of study outcomes.

#### Protocols

These form a substantial proportion of our publications, with randomised trials accounting for around half of our published protocols. We receive and publish protocols for a wide range of other designs, mainly mixed method, qualitative and quasi-experimental studies, and for programmes of research. Protocol publication increases transparency and acts as a guard against selective reporting, making it obvious if the research findings that are published differ from what was initially planned [18]. Also, by providing information about research in progress, protocol publication may reduce unplanned duplication of effort and encourage greater information sharing and collaboration worldwide.

We usually accept (without further peer review) protocols that have been through competitive peer review to receive funding from a nationally or internationally recognised research agency and that have received ethical review board approval or exemption. Protocols for programmes of research may be an exception to this requirement and are considered on a case-by-case basis.

We do not usually accept:

- Protocols that have not been the subject of peer review by a national or international research agency;
- Protocols for quality improvement or service evaluations, which lack a rigorous study design, such as uncontrolled before-after studies;
- Protocols for pilot studies. Because pilot studies are intended to lead on to subsequent, larger studies, there will be considerable overlap between the content of protocols for the two, and concerns about duplicate publication then arise. Authors should concentrate on publishing protocols for their subsequent, larger studies;
- Protocols for systematic reviews. We usually refer these to the BMC journal, *Systematic Reviews*;
- Protocols that are submitted for studies (particularly randomised controlled trials) where data cleaning and analysis have begun. Having a cut-off point like this is a common requirement of journals that publish trial protocols (in clinical trials, it is usually the end of patient recruitment) so that publication is a truly prospective event and the content of a protocol cannot be influenced (however unlikely this might be) by knowledge of the data. This may not apply to some qualitative studies but, in general, the intention is for a protocol to be published prior to any analysis in order to prevent bias.

Authors of implementation trial protocols also need to have registered the study with an appropriate trial database and to complete an appropriate reporting guideline checklist, such as the CONSORT extension to cluster randomised trials [19]. We also now encourage authors to ensure that protocol titles and abstracts include clearly labelled study designs which can assist further evidence syntheses.

#### Methodology

We welcome articles that present methods, which may either be completely new, or offer an improvement to an existing method. We favour submissions reporting empirical comparisons of one or more methodological approaches or which clearly state what they add to existing literature. We usually reject descriptive accounts of largely established methods, such as manuscripts reporting the development of a specific clinical guideline without any associated novel methodological insights.

#### Debate

We recognise the importance of debate and dialogue to move the field of implementation research forward. We welcome articles which question or challenge existing policies, practices, evidence or theory and suggest modifications or alternatives. However, as with other articles, we expect authors to demonstrate how they build upon existing bodies of knowledge or methodology. We often reject debate manuscripts which do not clearly articulate these, as well as those that do not fully contextualise their discussion in the existing implementation research literature.

Within this category, we are interested in publishing educational articles, particularly those that can inform early career researchers about key conceptual and methodological issues and therefore help develop capacity in our field. We plan to publish a number of educational articles for practitioners of implementation.

#### Short reports

We welcome brief reports of data from original research which present relatively modest advances in knowledge or methods. However, we usually do not publish meeting reports.

### Scope and boundaries related to article content

#### Potential significance of contribution to the literature

As highlighted earlier, we reject a large number of studies without peer review because they do not clearly articulate their contribution to current evidence or appear to make relatively minor contributions. This can be a difficult judgement but is a task made easier for editors and readers if authors clearly articulate what is already known and what their work adds to existing knowledge, theory and thinking. We plan to introduce a requirement that authors include such an explicit summary statement for all research papers.

We aim to treat effectiveness studies equally regardless of whether they report effects or no effects. We further recognise the importance of attempts to replicate previous findings, an objective which is often not sufficiently valued in the dash for novelty. We therefore welcome replications of implementation research accompanied by a clear rationale and will tend to reject those which are not.

We welcome studies on highly important but neglected topics in healthcare or population health which make more modest contributions to implementation research. We are likely to reject submissions focused on a narrow clinical domain or specific healthcare setting without a convincing case for wider generalisation.

#### End-points of interest

If studies evaluating the effects of implementation interventions are to be of relevance to policy and practice, they should have end-points related to evidence-based processes of care, patient outcomes or population outcomes. We receive submissions, sometimes as randomised trials, which report proxy end-points, such as beliefs and attitudes or unreliable measures such as self-reported measures of professional practice. Such end-points can have limited or low predictive validity; in other words they may not lead to or represent changes in actual behaviour. Naturally, similar measurement errors may be true of other end-points, such as processes of care or patient outcomes, which may not adequately capture the evidence-based behaviour of interest. For example, an increase in antidepressant prescribing does not necessarily represent better clinical practice nor improve patient outcomes—indeed, the opposite may be true. Authors, therefore, need to make the case, or provide evidence for, the validity of study end-points. We will however consider studies with proxy end-points presented as part of a programme of intervention development work and which recognise the need for further evaluation, such as explicitly theory-driven antecedents of clinical behaviour. We are likely to reject studies reporting proxy end-points which draw erroneous or far-fetched conclusions about likely ‘real world’ effects or are presented as standalone evaluations.

#### Population health interventions

We continue our interest in population (or public) health papers evaluating the effectiveness of the introduction of health-related practices of known effectiveness. To date, we have usually limited this to public health interventions involving healthcare settings or healthcare professionals. However, the boundaries can often become indistinct around, say, interventions targeting trained lay health workers or interventions in non-health settings, such as educational institutions. We further recognise the importance of and our responsibility to promote good science in improving population health. We therefore welcome submissions on the scaling-up of evidence-based population health interventions mediated through agencies, technologies or networks, typically involving healthcare providers but potentially others. We are also interested in implementation questions focused on national and local decision-makers and the implementation of interventions which connect people to resources in the community (e.g. third sector). We are likely to reject submissions on population health interventions where effectiveness has not been demonstrated. We recognise that the boundaries between what we consider to be implementation research and relatively more upstream population health research will remain challenging to distinguish. We will monitor this modification of scope and suggest alternative journals to authors of papers that we consider add more to fields other than implementation research.

#### Patient-mediated interventions

We have had a long-running debate within our editorial team on the boundaries around patient-mediated interventions [5]. We have hitherto welcomed studies in which patients may directly influence the behaviour of healthcare professionals in attempts to promote evidence-based practice. We have been less certain about studies of implementation interventions aimed directly at patients. This is partly because it becomes increasingly difficult to draw boundaries around such approaches which can, in effect, be extensions of clinical or health promotion interventions. However, if patients are acknowledged as key partners in healthcare, they can logically also be the target of implementation of evidence-based practice. We further acknowledge that many interventions mainly targeted at patients still require professional support [20]. Therefore, we are interested in implementation strategies directly targeting patients which also depend upon support from or co-management with healthcare professionals. We are less interested in studies exclusively focusing on influencing patients without professional involvement. We will actively keep this extension of scope under review and monitor the boundaries around studies of predominantly clinical or health-promoting interventions.

Table 1 summarises issues that influence the likelihood of rejection without review of articles submitted to *Implementation Science*.

**Table 1:** **Summary of issues that influence the likelihood of rejection without review of articles submitted to** ***Implementation Science***

**Issue**	**Likely to be accepted**	**Likely to be rejected**
Potential significance	Work contextualised within existing implementation research literature	Work not contextualised within existing implementation research literature
	Contribution to implementation research articulated and potentially significant	Contribution to implementation research not articulated or relatively minor
Field of interest	Healthcare and population health	Anything else
Effectiveness studies	Evaluating the effectiveness of implementation of an evidence-based practice or policy, or de-implementation of those demonstrated to be relatively ineffective or even harmful	Evaluating the effectiveness of a clinical, organisational, public health or policy intervention
Outcome	Health or health-related	Anything else
Implementation	Researching implementation	Doing implementation
Validity	Maximises internal and external validity as appropriate in the chosen study designs	
Patient decision aids	Evaluations of the implementation of patient decision aids (of known effectiveness) into healthcare settings; involvement of healthcare providers	Initial development, pilot testing or evaluation of patient decision aids
Implementation direct to patients	Outcomes referring to evidence-based practice with some involvement of healthcare providers	Other types of outcomes
Intervention development reports	Prepared and submitted prior to the reporting of the effectiveness of the intervention	*Post hoc* submission
	Going to be, (robustly) evaluated	Not going to be (robustly) evaluated
	Providing empirical and/or theoretical rationale	
Process evaluation	Submitted contemporaneously with or following report of intervention effectiveness	Process evaluations submitted in advance of the conduct of the main effectiveness analysis (it cannot be clear if they are explaining an effect or the absence of an effect)
	Process evaluations that take account of the main evaluation outcomes	Process evaluations that do not take account of the main evaluation outcomes
Pilot studies	If appropriate criteria for conduct	No justification for conduct
	If appropriate degree of inference	Overclaim on basis of results
	If there are plans for further evaluation	
Protocols	Been through peer review by a nationally recognised research agency as part of their funding	Not been through peer review by a nationally recognised research agency as part of their funding
	Received ethics review board approval	Not received ethics review board approval
	Submitted prior to data cleaning or analysis	Have begun data cleaning or analysis (may not apply to some qualitative studies)

### Current ambitions

Given the excellent submissions we receive, we are confident that *Implementation Science* will continue to play an important role in advancing the field. We have identified some specific needs and have published editorials welcoming further manuscripts on de-implementation and economic evaluations [9,20]. We are keen to promote research in low- and middle-income countries and in important but under-researched topics. We also wish to publish more educational articles covering conceptual and methodological issues which can promote capacity development and more rigorous research.

We are grateful to our authors, reviewers and editors who have contributed to our growth. We recognise that we do not always get it right and hope to improve the quality of our reviews and speed of turnaround. We will continue to review and refine our policies as the journal and the research field continue to evolve. We welcome author and reader comments (submitted via the ‘Comment’ facility associated with our articles) and debate to guide our future development.
